# 1428. The proportion of excess hospital-onset antibiotic-resistant infections attributable to patients diagnosed with COVID-19 in U.S. hospitals, 2019-2021

**DOI:** 10.1093/ofid/ofad500.1265

**Published:** 2023-11-27

**Authors:** Hannah Wolford, Natalie McCarthy, James Baggs, Sujan Reddy

**Affiliations:** CDC, Atlanta, Georgia; CDC, Atlanta, Georgia; CDC, Atlanta, Georgia; CDC, Atlanta, Georgia

## Abstract

**Background:**

Hospital-onset (HO) antibiotic-resistant (AR) infections increased in 2020. We examined what proportion of HO AR infection increases could be attributed to patients with COVID-19.

**Methods:**

HO cases were identified using PINC-AI microbiology data from a cohort of 240 hospitals in 2019-2021. The data were divided into a pre-pandemic period (Jan 2019-Jan 2020) and a pandemic period (Apr 2020-Dec 2021). We built a predictive model for HO AR infections using multivariable logistic regression including patient and facility demographics. The predictive model was built using pre-pandemic period data and applied to pandemic period patients not diagnosed with COVID-19 and pandemic period patients with COVID-19 (COVID cohort). The excess number of HO AR infections (observed – expected) was determined for each cohort and the proportion of excess attributable to the COVID cohort was calculated. We then repeated the predictive model process adding patient clinical characteristics in the model. Recycled predictions were used to estimate confidence intervals.

**Results:**

Before adjusting for clinical characteristics, MRSA had the highest proportion of excess HO AR cases attributable to the COVID cohort (100%; 95% CI 100-100%). ESBL (49%; 95% CI 45-53%), CRAsp (48%; 95% CI 44-54%), MDR *Pseudomonas* (48%; 95% CI 43-54%) and CRE (52%; 95% CI 45-61%) all had similar proportions of excess (Table). VRE had the lowest proportion of excess (24%; 95% CI 20-29%). After adjusting for clinical characteristics, the proportion of excess attributable to the COVID cohort decreased to 0% for MRSA, CRE, and VRE. CRAsp had the highest proportion of excess after adjusting (40%; 95% CI 35-45%); ESBL (15%; 95% CI 10-21%) and MDR *Pseudomonas* (8%; 95% CI 0-15%) had lower but non-zero proportions of excess. Rates of excess cases attributable to the COVID cohort can be seen in the Figure.

**Table.**
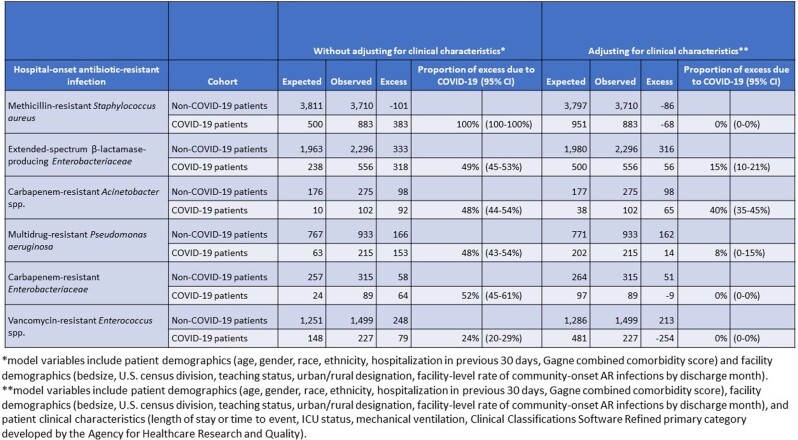
Model results with and without adjusting for patient clinical characteristics of the observed and excess hospital-onset (HO) antibiotic-resistant (AR) cases stratified by patients with and without COVID-19 as well as the proportion of excess HO AR cases attributable to patients diagnosed with COVID-19 (with 95% confidence intervals), by pathogen.

**Figure.**
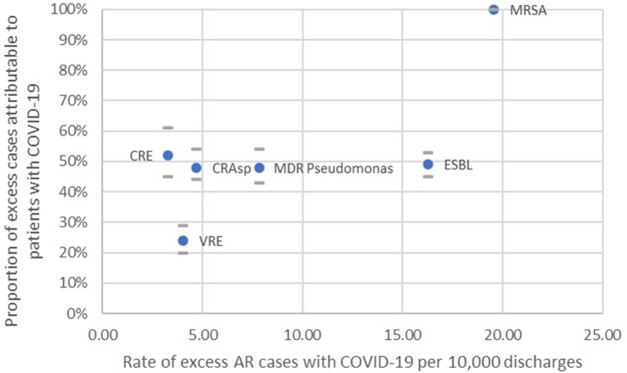
Comparison of the rates of excess hospital-onset (HO) antibiotic-resistant (AR) cases diagnosed with COVID-19 per 10,000 COVID-19 discharges to the proportion of excess HO AR cases attributable to patients diagnosed with COVID-19 (with 95% confidence intervals) for 6 pathogens, methicillin-resistant Staphylococcus aureus (MRSA), vancomycin-resistant Enterococcus spp. (VRE), carbapenem-resistant Enterobacteriaceae (CRE), extended-spectrum β-lactamase (ESBL)-producing Enterobacteriaceae, carbapenem-resistant Acinetobacter spp. (CRAsp), and multidrug-resistant Pseudomonas aeruginosa (MDR Pseudomonas) without adjusting for clinical characteristics.

**Conclusion:**

Patients with COVID-19 accounted for 20-100% of excess HO AR infections, varying by pathogen. In COVID-19 patients, increases in HO AR infections were largely due to patients’ clinical characteristics such as length of stay and comorbidities. In non-COVID-19 patients, increases in HO AR infections were not entirely explained by clinical characteristics, and might be due to factors such as lapses in infection prevention practices.

**Disclosures:**

**All Authors**: No reported disclosures

